# Sandalwood Oil and Turmeric-Based Cream Prevents Ionizing Radiation-Induced Dermatitis in Breast Cancer Patients: Clinical Study

**DOI:** 10.3390/medicines4030043

**Published:** 2017-06-24

**Authors:** Suresh Rao, Sanath Kumar Hegde, Manjeshwar Poonam Baliga-Rao, Jyothi Lobo, Princy Louis Palatty, Thomas George, Manjeshwar Shrinath Baliga

**Affiliations:** 1 Department of Radiation Oncology, Mangalore Institute of Oncology, Mangalore 575002, India; raos_64@yahoo.com (S.R.); Sanathkumarhegdemio@yahoo.com (S.K.H.); 2 Pharmacy Division, Mangalore Institute of Oncology, Mangalore 575002, India; poonam.baliga.rao@gmail.com; 3 Nursing Division, Mangalore Institute of Oncology, Mangalore 575002, India; jyothilobo.mio@gmail.com; 4 Department of Pharmacology, Father Muller Medical College, Kankanady, Mangalore 575002, India; drprincylouispalatty@gmail.com (P.L.P.); jeffthomasgeorge@gmail.com (T.G.)

**Keywords:** Vicco turmeric, breast cancer, radiation, radiodermatitis

## Abstract

**Background**: The primary objective of this study was to ascertain the benefit of Vicco turmeric Ayurvedic cream (VTC; Vicco Laboratories, Mumbai, India) sandalwood oil and turmeric-based cream in preventing radiodermatitis in women undergoing curative radiotherapy for their breast cancer. **Methods and Materials**: The study was an investigator-blinded randomized study with Johnsons Baby Oil (JBO; Johnson & Johnson Ltd., Baddi, India) as a comparator, administered daily from the start of radiation therapy for 5 weeks in women receiving breast radiation therapy, 50 Gy in 2 Gy fractions daily for 5 weeks. The endpoints were to ascertain the delay in the appearance and the degree of severity of dermatitis throughout the study period in accordance to the Therapy Oncology Group (RTOG) score. **Results**: The results indicated that the topical application of VTC delayed and mitigated the radiodermatitis. When compared to the Johnson’s Baby Oil, a significant decrease (*p* = 0.025) in the incidence of grade 1 was seen at week two, and also in grade 2 and 3 at week 3 (*p* = 0.003) and week 4 (*p* = 0.02), respectively, in the VTC cohort. A concomitant decrease in the average severity was also observed at week 2 (*p* = 0.02), week 3 (*p* = 0.05) and week 4 (*p* = 0.03). Conclusions: The results indicate that VTC cream significantly reduces radiation dermatitis when applied to the breast during and after radiation therapy. The result of this study indicates the beneficial effects. Double blind randomized control studies are required to further confirm the beneficial effects of VTC in mitigating radiodermatitis is people undergoing radiation treatment for their cancer.

## 1. Introduction

Globally, breast cancer is the leading cancer in women, and depending on the clinical stage and histopathological profile, to decrease the loco-regional recurrence and improve overall survival after surgery (conservative or radical mastectomy) and chemotherapy, patients are mostly treated with radiotherapy [1]. However, the major shortcoming of using radiation therapy is the development of radiation dermatitis, and its severity depends on intrinsic factors like age, general health, ethnic origin, hormonal status, genetic predisposition, basal skin conditions, co morbidities, pre-existing connective tissue and/or autoimmune disorders, and skin phototype; while extrinsic factors like radiation dose, volume, and number of fractions of radiation play a role [2]. Dermatitis is associated with pain, warmth, burning, and itching, and may impair quality of life and the treatment schedule, thereby possibly being prejudicial to the effective local control of the tumor cells [2].

Mechanistically, low linear energy transfer (LET) energy sources (like X-ray, gamma irradiation) commonly used in treating cancer predominantly mediate their cytotoxic effects by indirect effects through generating surplus free radicals resulting from radiolysis of water [3]. These free radicals subsequently cause damage to macromolecules like DNA, proteins and lipids [3]. Exposure to ionizing radiation also stimulates transcriptional activation of a cascade of cytokines, like tumor necrosis factor alpha (TNF-α) and Interleukin 1 (IL-l), to activate inflammatory response [4]. Radiation also modulates communication between cells, changes the skin’s endothelial cells, and triggers skin cell death, thereby affecting repopulation by damaging the proliferating stem cells present in basal layer of the dermis [4]. Cumulatively, all of these events impair cutaneous integrity and culminate in skin damage as characterized by swelling, redness, pigmentation, fibrosis, and ulceration of the skin [2].

Radiodermatitis is managed using conventional skin and wound care products, like use of steroidal, non-steroidal and metallic topical preparations and dressings [2,5]. Vast variation in the choice of agent and of treatment protocols exists among treating hospitals, and at times the choice of treating physician also varies [5]. Recently, herbal-based drugs have also been used, and scientific reports indicate that Aloe vera [6], silymarin [7] and marigold [1] based cosmecutical creams are beneficial. In our previous study, it was observed that Vicco turmeric cream (VTC), a turmeric (*Curcuma longa* L.) and Sandalwood oil (Santalum album L) based cream (containing turmeric extract 16% *w/w*, Sandalwood Oil 0.5% *w/w* in a non-greasy base) was effective at mitigating radiodermatitis head and neck cancer patients undergoing curative radiotherapy [8]. VTC has been used to ameliorate radiation-induced dermatitis anecdotally, but there is a lack of scientific data to support its use. In this endeavor, we attempted to ascertain whether VTC was effective in preventing radiodermatitis in women undergoing curative radiotherapy for their breast cancer with a parallel comparator group using Johnson’s Baby Oil (JBO).

## 2. Materials and Methods 

### 2.1. Patients and Methods

Sample size selection: The sample size was selected using the following formula; Where *p*_1_ = 0.2; *p*_2_ = 0.75; *p* = 0.2 + 0.75 ÷ 2; *Z_α_* = 1.96 at 95% confidence interval; *Z_β_* = 1.28 at 90% power to give a sample size of 18 in each group. Considering possible attrition, we rounded up the number to twenty subjects in each cohort.

(1) $$N=\frac{2{\left(Z_{\alpha }+Z_{\beta }\right)}^{2}p(1-p)}{p_{1}-p_{2}},$$

### 2.2. Patients and Randomization

This was a prospective study and was carried out between July 2013 and December 2013 in the Department of Radiotherapy at Mangalore Institute of Oncology, Mangalore, India. The study was done only with women who were between 18 and 80 years of age, with confirmed diagnosis of unilateral breast cancer with no invasive or metastatic cancer; who had had radical mastectomy followed by adjuvant chemotherapy; were in the inpatient facility throughout the radiation treatment; had no history of prior RT to the chest wall, no history of any connective tissue disorder or rashes or unhealed wounds in the radiation field; required >45 Gy of radiotherapy with no use of bolus or concurrent chemotherapy; and had a Karnofsky Performance Scale of above 70 (patient can care for self but is unable to carry on normal activity or to do active work). 

The exclusion criteria included patients less than 18 years of age; women with positive pregnancy test, on high doses of non-steroidal anti-inflammatory drugs; with significant co morbidity diabetes mellitus; undergoing radiation treatment on an out-service basis; confirmed cases of mental illness (like schizophrenia, bipolar disorders and severe depression) prior to discovery of cancer; and who developed hypersensitivity for the JBO or VTC cream following a patch test on the back. The eligible volunteers were then included in the study and randomized into either of two groups using opaque envelopes by an investigator unaware of the patient’s details. Patients allocated to group one received JBO oil, while group two received the VTC. The study was initiated and carried out after obtaining the approval of the institute’s ethics committee and in agreement with the guidelines of Helsinki declaration for research with humans.

### 2.3. Radiation Therapy Treatment

All patients who participated in this study received external irradiation from a linear accelerator (Varian, Model Unique Performance, Palo Alto, CA, USA) at a maximum energy level of 6 MV at a dose rate of 300. All planned fields were treated every day and depending on the clinical condition a conventional treatment schedule (2 Gy of IR per day, five times a week without any intended gaps for a planned target dose of 50 Gy for five consecutive weeks).

### 2.4. Application of JBO or VTC

Before the start of radiation, the volunteers and their caregivers were also taught the correct way of applying the JBO (5 mL) or VCT (5 g) by one of the investigators. The application of both JBO and VTC were five times a day (two hours before, immediately after, two hours after; four hours and six hours after radiotherapy) with the ventral surface of the fingers using a rotary motion with light pressure to the skin. The creams were massaged into the skin until the surface of the skin no longer felt greasy. To preserve the single blinding, patients were instructed not to use the agent 2 h or less before an irradiation session or before the treatment evaluation. No other prophylactic creams, lotions, or gels were allowed during the study period.

The patients of both cohorts were advised to use lukewarm water to wash, and with gentle detergent; not to use hair shampoo, not rub or scratch irradiated skin, pat skin dry with a soft towel after washing and to keep irradiated skin dry. During the course of the treatment, one of the investigators checked for the usage of the cream/oil on a weekly basis and repeatedly instructed the volunteers of both arms to adhere to the application of oil/cream. As one point of application was immediately after irradiation and all patients were residing in the inpatient services during the whole of the radiation treatment, it was easy for the investigators to keep a record of the patient’s adherence to oil/cream.

### 2.5. Diet of the Patients

The diet plan was planned in accordance to the stipulated requirement by a trained dietitian. As most of the patients were used to a rice-based diet, the hospital provided them with the standard diet, which mostly consisted of konji (mashed rice) seasoned with cooked vegetables and green gram or yellow gram. They were also provided with either vegetable soup or chicken soup, depending on their preference. Rice, ragi stew, wheat based breakfast and boiled eggs were also provided. In addition to this, they were also provided with dietary supplements ProSure (Abott Laboratories, SA, Camino de Purchil, Granda, Spain) three times a day as an additional source of protein during the treatment period under the supervision of a cancer dietitian and a trained nurse. The primary caregivers were requested to monitor the volunteer’s diet and medication when prescribed.

### 2.6. Patient Evaluation

The dermatological analysis was undertaken once weekly in accordance with the criteria of the Radiation Therapy Oncology Group/ European Organization for Research and Treatment Cancer (RTOG/EORTC) [9] as depicted in Table 1 by a senior radiation oncologist. The criteria were as follows: Grade 0: no skin rending, ulceration, inflammation or damage; Grade 1: faint erythema or dry desquamation; Grade 2: moderate to brisk erythema, patchy moist desquamation mostly confined to skin folds and creases, moderate edema; Grade 3: radiation dermatitis consists of moist desquamation ≥1.5 cm diameter, other than skin folds or creases and bleeding induced by minor trauma or abrasion; and Grade 4: skin necrosis or ulceration of full thickness dermis; spontaneous bleeding from the involved site. This score makes a useful distinction between faint erythema and tender, bright erythema, as well as between patchy and confluent moist desquamation, and is probably the most widely used in practice and research [1,2]. On every investigation, the investigator able considered the score for the worst toxicity in the treatment field. As one single investigator graded the dermatitis there was no need for standardization. 

### 2.7. Statistical Analysis

The data from individual patients were noted down in a separate book and entered in to the Microsoft Excel. The demographic and patient specific data were subjected to frequency. The χ^2^ test was used to compare the age, treatment dose and incidence of dermatitis; while student’s “*t*” test was used to ascertain the difference in the degree of dermatitis at all the time points. A *p* value of < than 0.05 was considered significant.

## 3. Results

Demographic details like age, religion and the details on type and stage of cancer of the available patients are represented in Table 1. With respect to age and religion, there was no statistically significant difference, and this is represented in Table 1. An important aspect that was observed is that some of the patients did not have their tissues sampled for the immunohistochemical analysis of ER, PR and Her/Neu2, and that most of them had come in for the radiation treatment after completing their surgery and chemotherapy in another hospital.

The radiation dermatitis was evaluated in accordance to the RTOG guidelines, and at the end of first week patients of both arms did not show any symptoms of radiodermatitis. With continuation of the radiation at the end of the second week 75% (15/20) and 32% (8/20) of volunteers in the JBO and VCT groups, respectively, developed dermatitis, which was statistically significant (χ^2^; *p* = 0.025). The incidence of total number of individuals with dermatitis increased with the radiation dose and some volunteers also developed grade 2 and 3 in both the groups. However, their numbers were less in the VTC than in the JBO group, and were statistically significant at week 3 (χ^2^; *p* = 0.003) and week 4 (χ^2^; *p* = 0.02). 

**Figure 1:**
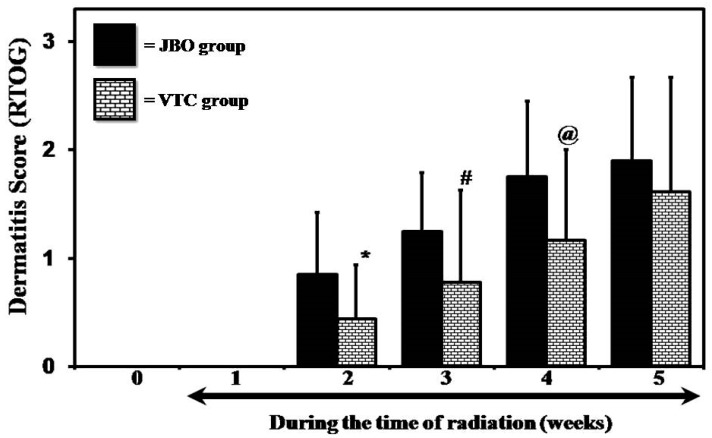
Different in the degree of radiation-induced dermatitis (Student’s “*t*” test * is *p* < 0.02; # is *p* < 0.05 and @ is *p* < 0.03).

At the end of the study, it was observed that no patients developed grade 4 dermatitis in either of the groups, or also that 5% (1/20) and 15% (3/20) of the patients in the JBO and VTC did not develop any dermatitis. The average dermatitis developing throughout the five-week study period also showed that the application of VTC was effective in reducing the degree and severity, and was statistically significant at week 2 (“*t*” test; *p* = 0.018), week 3 (“*t*” test; *p* = 0.05) and week 4 (“*t*” test; *p* = 0.03) (Figure 1).

## 4. Discussion

Radiation dermatitis is an important side effect, and data indicate it to be common in nearly 80% of all patients during the course of the treatment [1]. In this study, it was observed that the VTC cream was more effective at delaying the incidence and also the degree of dermatitis. These observations are in agreement with earlier observations where VTC cream was observed to be better than Johnson’s baby oil at mitigation of radiodermatitis in head and neck cancers [8]. The results of this study show that the intensity of dermatitis was decreased in the VTC group, and also that there was a delay in the appearance of dermatitis for grade 1, 2 and 3, indicating it to be effective. On a comparative note, VTC was beneficial in both breast and head and neck [8], indicating that the cream was effective at mitigating radiation dermatitis in different anatomic sites. Additionally, studies have also shown that oral intake of 2.0 grams of curcumin three times per day (i.e., 6.0 grams daily) during radiotherapy reduced the severity of radiation dermatitis in breast cancer patients [10]. 

In the Ayurvedic system of medicine, both turmeric and sandalwood have a long history of being useful for various skin ailments, and experiments have validated their ethnomedicinal uses [11]. VTC composed of turmeric and sandalwood is arguably one of the oldest cosmecutical creams, and has been marketed in India for over 40 years [8]. These plants, as well as their principal constituents curcumin and santalol, have been shown to be beneficial in skin care [8]. From a mechanistic perspective, studies have shown that turmeric/curcumin possess chemopreventive properties and is found to be beneficial in treating various disorders, including skin diseases [12]; to possess free radical scavenging effects [11,13,14]; and to decrease radiation-induced lipid peroxidation [15] and DNA damage [15,16]. Turmeric and curcumin also possess anti-inflammatory effects on skin in various experimental models [17], and to mediate these effects by inhibiting induction of ornithine decarboxylase activity, DNA synthesis, epidermal lipoxygenase and cyclooxygenase activities, inhibiting activity of ERK and activation of NF-κB [11,12,14,18,19,20,21]. 

Turmeric/curcumin have been shown to enhance wound healing against various conditions [11], and mechanistic studies have shown this to be mediate by increasing re-epithelialization of the epidermis; migration of myofibroblasts, fibroblasts and macrophages to the wound bed; promotion of neovascularization; and, by greater deposition of collagen in rats, increasing levels of TGF-β1 [22]. To further substantiate, recent studies with diabetic rats have also shown that curcumin was effective at enhancing open excisional diabetic wounds and mediating this by increasing VEGF and TGF-β1, hypoxia-inducible growth factor-1 alpha, stromal cell-derived growth factor-1 alpha, and heme oxygenase-1 [23]. 

Curcumin has also been shown to promote open wound healing in mice exposed to whole body and hemibody radiation, and this effect was mediated by enhancing wound contraction, collagen synthesis, increasing hexosamine, DNA synthesis and improving fibroblast and vascular densities [24,25,26,27]. Molecular studies with laboratory mice exposed to radiation have also shown that administering curcumin markedly reduces the acute and chronic skin toxicity and mediates beneficial effects by decreasing the mRNA expression of early responding cytokines (IL-1, IL-6, IL-18, TNF-α and lymphotoxin-β) and the fibrogenic cytokine, TGF-β, in cutaneous tissues [21]. Considering all these observations, it can be postulated that the beneficial effects of VTC in preventing radiation-induced dermatitis can be best explained by its anti-inflammatory, antioxidant, modulating cytokines and by enhancing wound healing process, and that a complementary mechanism may mediate the protective effects. Mechanistic studies are being planned to unravel the protective pathways being triggered/involved in observed protective effects.

## 5. Conclusions 

The results of the present study indicate the usefulness if VTC in preventing the radiation induced dermatitis in women undergoing treatment for their breast cancers. These effects can be best explained by its anti-inflammatory, antioxidant, modulating cytokines and by enhancing wound healing process, and that a complementary mechanism may mediate the protective effects. Mechanistic studies are being planned to unravel the protective pathways being triggered/involved in observed protective effects.

## Figures and Tables

**Table 1:** Patient and tumor characteristics.

Details	JBO (Johnsons Baby Oil) (20)	VTC (Vicco Turmeric Cream) (20)
Age (in years)	49.11 ± 10.1	50.93 ± 9.52
Religion	JBO (20)	VTC (20)
Hindu	13	14
Muslim	5	3
Christian	2	3
Histologic type of tumor	JBO (20)	VTC (20)
Infiltrating ductal carcinoma	17	18
Infiltrating lobular carcinoma	3	2
TNM stage (20/20)	JBO (20)	VTC (20)
Primary		
T_1_	1	1
T_2_	6	8
T_3_	8	
T_4_	5	
Regional nodes	7	VTC (20)
N_0_	4	4
N_1_	8	5
N_2_	5	9
N_3_	2	2
Immunohistochemistry	JBO (14)	VTC (11)
ER–/PR–	2	3
ER+/PR–	7	5
ER+/PR+	5	3
HER2-positive	10	8
HER2-negative	4	3
No reports	6	9
Radiation treatment details	JBO (20)	VTC (20)
Dose of radiation (Gy)	50	50
Fraction (2 Gy × 5 days a week × 5 weeks)	25	25
Healthy breast tissue volume (cc)	639.26 ± 359.63	627.51 ± 258.32
Healthy breast tissues received dose (Gy)	30.76 ± 16.91	26.82 ± 14.82
Skin dose at treated breast (Gy)	48.61 ± 15.43	49.24 ± 17.93
